# Cleavage of 14-3-3ε by the enteroviral 3C protease dampens RIG-I-mediated antiviral signaling

**DOI:** 10.1128/jvi.00604-23

**Published:** 2023-08-09

**Authors:** Daniel D. T. Andrews, Marli Vlok, Dorssa Akbari Bani, Brenna N. Hay, Yasir Mohamud, Leonard J. Foster, Honglin Luo, Christopher M. Overall, Eric Jan

**Affiliations:** 1 Department of Biochemistry and Molecular Biology, University of British Columbia, Vancouver, British Columbia, Canada; 2 Life Sciences Institute, University of British Columbia, Vancouver, British Columbia, Canada; 3 Michael Smith Laboratories, University of British Columbia, Vancouver, British Columbia, Canada; 4 Department of Pathology and Laboratory Medicine, University of British Columbia, Vancouver, British Columbia, Canada; 5 Centre for Heart Lung Innovation, University of British Columbia, Vancouver, British Columbia, Canada; 6 Department of Oral Biological and Medical Sciences, University of British Columbia, Vancouver, British Columbia, Canada; 7 Centre for Blood Research, University of British Columbia, Vancouver, Canada; Hudson Institute of Medical Research, Clayton, Victoria, Australia

**Keywords:** enterovirus, protease, innate immune response, RIG-I, antiviral, interferon

## Abstract

Viruses have evolved diverse strategies to evade the host innate immune response and promote infection. The retinoic acid-inducible gene I (RIG-I)-like receptors RIG-I and MDA5 are antiviral factors that sense viral RNA and trigger downstream signal via mitochondrial antiviral-signaling protein (MAVS) to activate type I interferon expression. 14-3-3ε is a key component of the RIG-I translocon complex that interacts with MAVS at the mitochondrial membrane; however, the exact role of 14-3-3ε in this pathway is not well understood. In this study, we demonstrate that 14-3-3ε is a direct substrate of both the poliovirus and coxsackievirus B3 (CVB3) 3C proteases (3C^pro^) and that it is cleaved at Q236↓G237, resulting in the generation of N- and C-terminal fragments of 27.0 and 2.1 kDa, respectively. While the exogenous expression of wild-type 14-3-3ε enhances *IFNB* mRNA production during poly(I:C) stimulation, expression of the truncated N-terminal fragment does not. The N-terminal 14-3-3ε fragment does not interact with RIG-I in co-immunoprecipitation assays, nor can it facilitate RIG-I translocation to the mitochondria. Probing the intrinsically disordered C-terminal region identifies key residues responsible for the interaction between 14-3-3ε and RIG-I. Finally, overexpression of the N-terminal fragment promotes CVB3 infection in mammalian cells. The strategic enterovirus 3C^pro^-mediated cleavage of 14-3-3ε antagonizes RIG-I signaling by disrupting critical interactions within the RIG-I translocon complex, thus contributing to evasion of the host antiviral response.

**IMPORTANCE:**

Host antiviral factors work to sense virus infection through various mechanisms, including a complex signaling pathway known as the retinoic acid-inducible gene I (RIG-I)-like receptor pathway. This pathway drives the production of antiviral molecules known as interferons, which are necessary to establish an antiviral state in the cellular environment. Key to this antiviral signaling pathway is the small chaperone protein 14-3-3ε, which facilitates the delivery of a viral sensor protein, RIG-I, to the mitochondria. In this study, we show that the enteroviral 3C protease cleaves 14-3-3ε during infection, rendering it incapable of facilitating this antiviral response. We also find that the resulting N-terminal cleavage fragment dampens RIG-I signaling and promotes virus infection. Our findings reveal a novel viral strategy that restricts the antiviral host response and provides insights into the mechanisms underlying 14-3-3ε function in RIG-I antiviral signaling.

## INTRODUCTION

In response to viral infections, mammalian cells have evolved several distinct mechanisms to sense and respond to the presence of a virus. Sensing of exogenous genetic material, proteins, and other viral markers through these mechanisms in part triggers an enhanced antiviral state in the cell, characterized by the expression of interferons (IFNs) and, subsequently, interferon-stimulated genes (ISGs) that restrict infection (1). In an “arms race” between virus and host, viruses must counter these mechanisms and, as such, have evolved intricate evasion mechanisms, such as the capping of viral genetic material to mimic cellular RNA, the sequestering of the replication complex to shield the viral particles from detection, and the proteolytic processing of cellular antiviral proteins (2 - 5).

Mammalian cells sense RNA virus infections through membrane-associated toll-like receptors and cytoplasmic retinoic acid-inducible gene I (RIG-I)-like receptors (RLRs), including RIG-I (which senses the 5′ ends of RNA, including 5′ triphosphate, duplex RNA, and no ribose 2′-O-methylation of 5′ terminal nucleotide), melanoma differentiation-associated protein 5 (MDA5, which senses long dsRNAs), and laboratory of genetics and physiology 2 (LGP2, which acts as a regulator for other RLRs) (6 - 8). In general, RLRs are activated upon the detection of viral dsRNA and RNAs containing a 5′ triphosphate moiety but no methyl cap, though MDA5 can also detect foreign RNAs lacking 5′ triphosphates (1). Upon detection of viral RNA, RIG-I and MDA5 undergo conformational changes from a “closed” to an “open” conformation (9). The closed conformation prohibits access to the Caspase Activation and Recruitment Domains (CARDs), while the open conformation exposes them. Access to the CARDs is dependent on several factors, such as ubiquitination of RIG-I with K63-polyubiquitin chains and the association of RIG-I with chaperone proteins such as tripartite motif containing 25 (TRIM25) and 14-3-3ε (10, 11). These interactions enable the CARDs of RIG-I to associate with the CARDs of mitochondrial antiviral-signaling protein (MAVS), which serves as an essential step in the RLR pathway (12). MAVS activation via the CARDs then triggers a downstream cascade that drives type 1 IFN production (6, 13). Notably, the activation of these pathways is virus-specific, with either RIG-I or MDA5 acting as the primary sensor for a given virus during infection (14).

A subset of 14-3-3 proteins plays key roles in the RIG-I and MDA5 signaling pathways. The 14-3-3 family of proteins consists of seven unique isoforms: β, γ, ε, ζ, η, σ, and τ, that function as regulatory molecules in cellular pathways such as cell death and apoptosis, cell cycle regulation, and the cellular antiviral response (15 - 21). 14-3-3 proteins adopt a conserved α-helical fold, in which a binding groove recognizes phosphorylated serine or threonine residues within an RSXpSXP or RXXXpSXP motif (16, 22). 14-3-3 interactions regulate protein function by inducing a conformational change, sequestering or relocalizing the target protein, or acting as a molecular scaffold (23 - 25). The primary differences between the various 14-3-3 proteins lie in the variable C-terminal region, the function of which is poorly understood. Liu et al. (11) identified 14-3-3ε as a key interaction partner of RIG-I during activation, demonstrating that 14-3-3ε binding is necessary for the translocation of RIG-I to the mitochondria, which is an essential step in its interaction with and activation of MAVS. In addition to RIG-I interactions with 14-3-3ε and TRIM25 (10), UFL1-mediated ufmylation is a key step in RIG-I translocation (26). In a comparable manner, highlighting the specificity of 14-3-3 proteins, 14-3-3η promotes MDA5 activation and translocation in hepatitis C virus-infected cells (15).

Several viruses have evolved countermeasures to this pathway, highlighting the importance of RLRs and 14-3-3 proteins in the cellular antiviral response. The NS3 protein of dengue and Zika viruses contains a phosphomimic domain that binds to and sequesters 14-3-3ε, antagonizing the RIG-I response and promoting viral replication (18, 20). The influenza A virus NS1 protein similarly interacts with 14-3-3ε and disrupts RIG-I translocation to the mitochondria (27). Epstein–Barr virus, Kaposi sarcoma-associated herpesvirus, and human cytomegalovirus all encode ubiquitin deconjugases that interact with 14-3-3 proteins to drive TRIM25 aggregation and inactivation, promoting infection and inhibiting IFN production (28). Despite being a key RIG-I regulatory factor that is targeted by several distinct viral families, the exact role of 14-3-3ε in RIG-I translocation has not been fully elucidated.

In this study, we showed that 14-3-3ε is cleaved during poliovirus and coxsackievirus B3 (CVB3) infection and that it is a direct target of the enteroviral 3C protease (3C^pro^) with cleavage occurring between amino acids Q236↓G237 of 14-3-3ε. We demonstrated that expression of the N-terminal cleavage fragment of 14-3-3ε (“3CN”) blocks RIG-I-mediated *IFNB* transcription. Further, we showed that 14-3-3ε 3CN cannot interact with RIG-I and thereby disrupts the translocation of the RIG-I complex to the mitochondria. Finally, we revealed that the expression of 14-3-3ε 3CN promotes productive CVB3 infection and counteracts *IFNB* production during virus infection. These results reveal a novel strategy by which enteroviruses restrict and evade the host innate immune response.

## RESULTS

### Cleavage of 14-3-3ε in poliovirus and CVB3-infected cells

**Fig 1:**
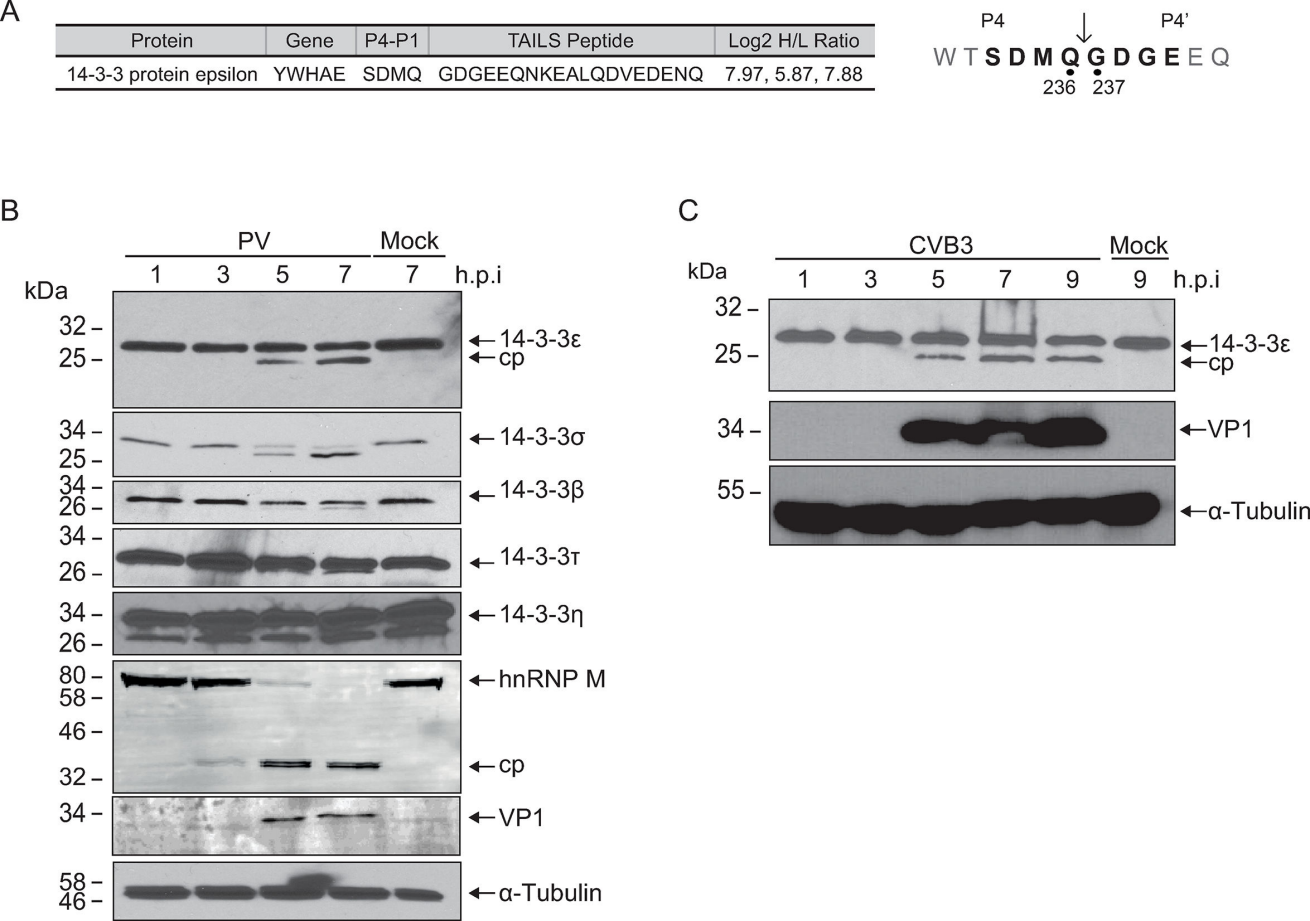
Cleavage of 14-3-3 family members in poliovirus and coxsackievirus B3 infection. (**A**) High-confidence 14-3-3ε cleavage peptide identified via TAILS analysis in poliovirus-infected Hela cells (MOI 10, 5 hours post-infection) (3). A peptide corresponding to 14-3-3ε was identified *via* mass spectrometry with the upstream P4-P1 and downstream P1′-P4´ sites indicated (*right*). Log_2_ H/L ratio was determined across three independent experiments (poliovirus-infected cells—heavy; mock infected—light). (**B**) Representative immunoblot of poliovirus-infected cell lysates (MOI = 10) infected for the indicated time point. Twenty micrograms of total lysate was separated on a 12% SDS-PAGE gel. (**C**) Representative immunoblot of CVB3-infected cell lysates (MOI = 10) infected for the indicated time point. Twenty micrograms of total lysate was separated on a 12% SDS-PAGE gel. cp, cleavage product.

Previously, we used Terminal Amine Isotopic Labeling of Substrates (TAILS), an unbiased N-terminal mass-spectrometry based approach, to identify host proteins that are cleaved by poliovirus and CVB3 3C^pro^ (3, 4, 29, 30). The TAILS data set (3) identified the high-confidence neo-N-termini peptide ^237^GDGEEQNKEA from human 14-3-3ε (Fig. 1A), a known chaperone protein and key regulator of the RIG-I activation pathway (11). Consistent with the preferred Q↓G (P1↓P1’) specificity of 3C^pro^ (31), the TAILS-identified cleavage site in 14-3-3ε is between Q236↓G237, prompting us to further investigate the functional role of cleavage of this protein.

First, we sought to determine whether 14-3-3ε is cleaved during virus infection. We infected HeLa cells with poliovirus and probed for 14-3-3ε by immunoblotting. A distinct, lower molecular weight band of 14-3-3ε cleavage product was observed 5 hours post-infection (h.p.i.) (Fig. 1B). The predicted cleavage event between Q236↓G237 would result in two protein fragments, an N- and C-terminal fragment of ~27.0 and ~2.1 kDa, respectively. To determine whether cleavage is specific to poliovirus or if it is a general strategy employed during enterovirus infection, we monitored the state of 14-3-3ε in CVB3-infected HeLa cells. Like that observed in poliovirus-infected cells, a cleavage product of 14-3-3ε was observed in CVB3-infected cells (Fig. 1C), suggesting a common viral cleavage strategy employed by enteroviruses.

We next examined whether other 14-3-3 family members are cleaved during infection. 14-3-3σ, β, and τ were also cleaved to varying extents during poliovirus infection (Fig. 1B). Like 14-3-3ε, cleavage fragments of 14-3-3σ and τ were detected as early as 5 h.p.i., while 14-3-3σ was cleaved to completion by 7 h.p.i. By contrast, no distinct 14-3-3η cleavage products were detected during infection using this antibody in these immunoblots. In summary, these results indicated that several 14-3-3 proteins are differentially cleaved to varying extents during poliovirus infection.

### Endogenous 14-3-3ε is cleaved directly by poliovirus 3C^pro^

**Fig 2:**
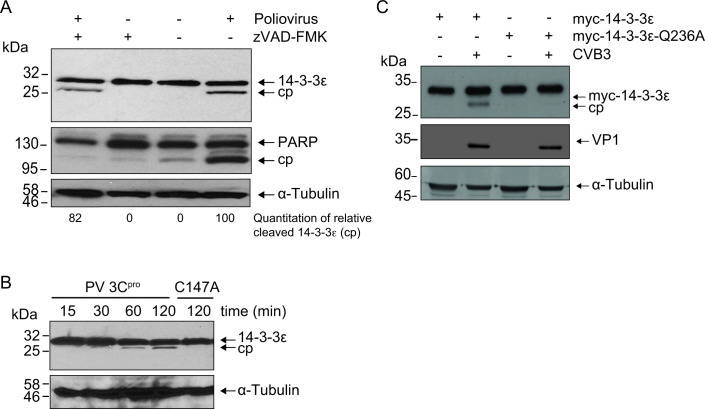
14-3-3ε is cleaved by poliovirus 3C^pro^. (**A**) Representative immunoblot of poliovirus-infected HeLa cells in the presence or absence of zVAD-FMK at 7 hours post-infection (h.p.i.) (MOI = 10). Below the blot is the amount of cleaved 14-3-3ε product (cp) relative to total 14-3-3ε (full-length and cleaved product combined, 100). (**B**) Representative immunoblot of an *in vitro* cleavage assay performed in HeLa cell lysates. One microgram of clarified cell lysate was incubated with either wild type (WT) or catalytically inactive (C147A) purified recombinant 3C^pro^ (100 ng/µL final concentration). (**C**) Representative immunoblot of lysates from CVB3-infected A549 cells transfected with either the WT 14-3-3ε or the cleavage-resistant mutant, Q236A (MOI = 10, 7 h.p.i.). cp, cleavage product.

14-3-3ε is a known substrate of caspase-3 during apoptosis, and apoptosis can be induced at late stages of poliovirus infection (21, 32, 33). To determine whether 14-3-3ε is targeted by caspases in poliovirus-infected cells, we pre-treated cells with zVAD-FMK, a pan-caspase inhibitor, and subsequently infected the cells with poliovirus for 7 hours. As expected, poly-(ADP-ribose) polymerase (PARP), a known substrate of caspase-3, was cleaved in poliovirus-infected cells but was not cleaved in the presence of zVAD-FMK (Fig. 2A). By contrast, 14-3-3ε cleavage was still detected in infected zVAD-FMK-treated cells, thus indicating that 14-3-3ε is still cleaved in the absence of caspase activity. Specifically, quantitating the relative amount of cleaved 14-3-3ε product in poliovirus-infected zVAD-FMK-treated cells compared to that in poliovirus-infected cells was ~82% (Fig. 2A, compare lanes 1 and 4). These results strongly indicated that the cleavage of 14-3-3ε in CVB3-infected cells is largely caspase-independent.

To determine whether 14-3-3ε is a direct substrate of 3C^pro^, we performed an *in vitro* cleavage assay using HeLa cell lysates incubated with either purified, recombinant wild type (WT) or catalytically inactive (C174A) poliovirus 3C^pro^. As expected, a cleavage fragment of 14-3-3ε, ~27 kDa, was detected in reactions containing purified WT, but not the mutant C147A 3C^pro^ (Fig. 2B). The ~27.0 kDa mass of the cleavage fragment was similar to that observed in poliovirus-infected cells (Fig. 1B and 2B).

The N-terminomics TAILS analysis identified cleavage of 14-3-3ε between Q236↓G237. To confirm this, we generated myc-tagged 14-3-3ε constructs expressing either the WT 14-3-3ε or a construct containing a glutamine to alanine mutation at the P1 site (Q236A). We transfected the WT or Q236A 14-3-3ε constructs into A549 cells and then infected the cells with CVB3. For subsequent experiments, we used A549 cells as opposed to HeLa cells due to the relevance of A549 cells as a model for virus infection and an intact innate immune response (34). Supporting our earlier observations, a cleavage product was detected in CVB3-infected cells expressing the WT but not the mutant Q236A 14-3-3ε (Fig. 2C). In summary, these results demonstrated that 14-3-3ε is a direct substrate of 3C^pro^ and that cleavage occurs at the Q236↓G237 site.

### Effects of the 14-3-3ε N-terminal cleavage product on cell viability

**Fig 3:**
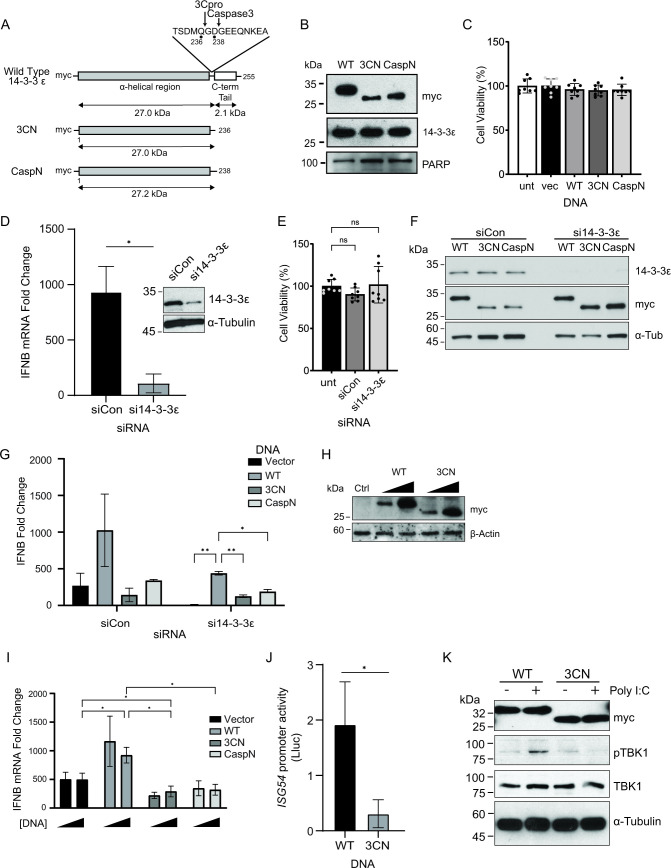
N-terminal 14-3-3ε dampens RLR-stimulated *IFNB* mRNA production. (**A**) Schematic of 14-3-3ε expression constructs. Wild type (WT) contains the N-terminal α-helical region (gray) and the C-terminal tail (white). Inset: amino acids flanking the 3C^pro^ cleavage site (Q236) and the caspase-3 cleavage site (D238). 3CN, 3C^pro^ N-terminal cleavage product (middle); CaspN, caspase-3 N-terminal cleavage product (bottom). (**B**) Immunoblot analysis of A549 cells transfected with either WT, 3CN, or CaspN 14-3-3ε for 24 hours. (**C, F**) Cell viability analysis. MTT assay of A549 cells transfected with the indicated DNA constructs (24 hours post-transfection). The percentage of viable cells in each well was determined by measuring absorbance at 580 nm. (**D**) RT-qPCR of *IFNB* mRNA from cells treated with either siRNA control (siCon) or si14-3-3ε for 24 hours and poly(I:C) for 6 hours. Expression levels were normalized internally to *GAPDH* and fold changes are relative to unstimulated control samples. Inset: immunoblot representation of lysates treated with siCon or si14-3-3ε for 24 hours. *N* = 3. (**E**) Immunoblot analysis of A549 cells treated with either siCon or si14-3-3ε for 24 hours and transiently transfected with the indicated DNA for a further 24 hours. (**G**) RT-qPCR of *IFNB* mRNA from cells treated with either siCon or si14-3-3ε for 24 hours, transfected with the indicated DNA for a further 24 hours, and stimulated with poly(I:C) for 6 hours. Expression levels were normalized internally to *GAPDH* and fold changes are relative to unstimulated control samples. *N* = 3. (**H**) Immunoblot analysis of A549 cells transfected with an increasing dose of either WT or 3CN 14-3-3ε overexpression constructs (100 ng or 500 ng) for 24 hours. (**I**) RT-qPCR of *IFNB* mRNA from cells transiently transfected with the indicated DNA for 24 hours and poly(I:C) for 6 hours. Expression levels were normalized internally to *GAPDH* and fold changes are relative to unstimulated control samples. *N* = 3. (**J**) Luciferase assay of A549-Dual cells transfected with the indicated DNA for 24 hours and transfected with poly(I:C) for 24 hours. The luminescence detected is the Lucia luciferase (Lluc) protein under the control of an ISG54 promoter and 5xISRE. *N* = 3. (**K**) Immunoblot analysis of A549 cells transfected with either WT or 3CN 14-3-3ε constructs for 24 hours and stimulated with poly(I:C) for 6 hours. *P < .05; **P < .005. A Welch’s *t*-test (**B, I**), a two-way ANOVA (**G, H**), or a one-way ANOVA (**E, F**) were used to analyze statistical significance.

A previous report showed that 14-3-3ε is targeted by caspase-3, which then leads to the release of BCL2-associated agonist of cell death to promote apoptosis (21). Interestingly, the caspase-3-mediated cleavage site occurs at D238, just two amino acids downstream of the Q236 cleavage site of 3C^pro^, and the caspase-induced N-terminal fragment plays a pro-apoptotic role (21). To determine whether the expression of these N-terminal cleavage fragments of 14-3-3ε has effects on cell viability, we generated a set of expression constructs containing a myc epitope tag at the N-terminal. Alongside the WT 14-3-3ε, we generated constructs that terminated at either Q236 (“3CN”) or D238 (“CaspN”) to mimic that of a stable cleavage fragment (Fig. 3A). Following transfection, we first assessed, by immunoblotting, caspase activity via PARP cleavage (Fig. 3B) (35). In all cases, the expression of the tagged 14-3-3ε proteins did not lead to detectable cleavage or loss of full-length PARP. Furthermore, overexpression of these 14-3-3ε proteins did not affect cell viability (Fig. 3C). Collectively, these results demonstrated that expression of the truncated 14-3-3ε protein fragments does not activate caspases or induce cell death in A549 cells.

### Effect of N-terminal 14-3-3ε expression on *IFNB* mRNA production

14-3-3ε is a key component of the RIG-I translocon complex, serving as a chaperone to promote RIG-I signaling and activate the downstream IFN response (26). We hypothesized that the cleavage of 14-3-3ε by 3C^pro^ and the resulting N-terminal fragment act to disrupt the RIG-I signaling pathway. First, to determine whether endogenous 14-3-3ε is necessary for *IFNB* mRNA production, we transfected an siRNA targeting the 3′ untranslated region (3′ UTR) of the *YWHAE* transcript of 14-3-3ε into A549 cells and confirmed a significant depletion of the endogenous protein (Fig. 3D, inset). To activate the RLR pathway, we transfected cells with the dsRNA analog polyinosinic:polycytidylic acid [poly(I:C)] and then measured *IFNB* mRNA levels by RT-qPCR. Transfection of poly(I:C) in the presence of a control siRNA targeting firefly luciferase resulted in an ~900-fold increase in *IFNB* mRNA levels compared to untreated cells, confirming activation of the RLR pathway. By contrast, poly(I:C) treatment of cells transfected with an siRNA targeting *YWHAE* significantly reduced the amount of *IFNB* mRNA (Fig. 3D), demonstrating the importance of 14-3-3ε in facilitating *IFNB* production, consistent with previously reported results (11).

The use of an siRNA targeting the 3′ UTR of the *YWHAE* transcript allows for exogenous expression of 14-3-3ε from transfected constructs, thus direct effects on the RLR signaling pathway can be assessed. Specifically, we utilized myc-tagged WT 14-3-3ε, or mutants containing truncated 14-3-3ε at the 3C^pro^ cleavage site Q236 (3CN) or the caspase cleavage site D238 (CaspN). Depletion of endogenous 14-3-3ε by siRNA treatment did not affect cell viability as compared to cells treated with control siRNA (Fig. 3E). Furthermore, tagged 14-3-3ε (Fig. 3A) was expressed in both control cells and cells depleted of endogenous 14-3-3ε by siRNA treatment (Fig. 3F). In cells depleted of endogenous 14-3-3ε, there was a significant increase in *IFNB* mRNA levels in WT expressing cells compared to the vector control, but no significant increase in cells expressing 3CN or CaspN compared to the vector control, suggesting that both truncated mutants did not enhance *IFNB* mRNA production (Fig. 3G).

We initially observed that only a subset of total endogenous 14-3-3ε was cleaved under virus infection (Fig. 1 and 2) and that full-length protein remained present throughout. Therefore, we next asked whether the N-terminal fragments could contribute to the dysregulation of *IFNB* signaling in the presence of the full-length endogenous protein. To investigate this further, we transfected each expression construct (100 or 500 ng) in cells for 24 hours followed by poly(I:C) stimulation (Fig. 3H), after which we measured *IFNB* mRNA levels by RT-qPCR (Fig. 3I). Compared to the vector control, the expression of the 3CN and CaspN, but not the WT 14-3-3ε or vector alone, resulted in a decrease in IFNB levels following poly(I:C) stimulation (Fig. 3I), suggesting that the truncated protein fragments are capable of reducing IFNB mRNA production in the presence of endogenous 14-3-3ε. To further confirm this observation, we monitored RLR signaling using an A549 cell line engineered to express an interferon-stimulated response element (5xISRE)-luciferase reporter. Overexpression of 3CN 14-3-3ε reduced relative luciferase activity compared to the WT 14-3-3ε, further confirming that expression of the N-terminal 14-3-3ε cleavage products can reduce RLR signaling in A549 cells (Fig. 3J).

The 14-3-3ε-containing translocon complex signals upstream of MAVS, which subsequently triggers TANK binding kinase (TBK1) phosphorylation (36). To determine the effects of 3CN 14-3-3ε expression on RLR signaling, we monitored TBK1 phosphorylation (p-TBK1) by immunoblotting. As expected, poly(I:C) treatment resulted in an increase in p-TBK1 levels in cells transfected with WT 14-3-3ε, indicating activation of the RLR signaling pathway (Fig. 3K). By contrast, expression of 3CN 14-3-3ε failed to promote TBK1 phosphorylation, indicating that expression of 3CN 14-3-3ε cannot activate the RLR signaling pathway upstream of TBK1 phosphorylation. These results demonstrate that the truncated N-terminal fragment derived from 3C^pro^-mediated cleavage of 14-3-3ε reduces overall RLR signaling.

### Key residues in the C-terminal tail of 14-3-3ε for RIG-I signaling

**Fig 4:**
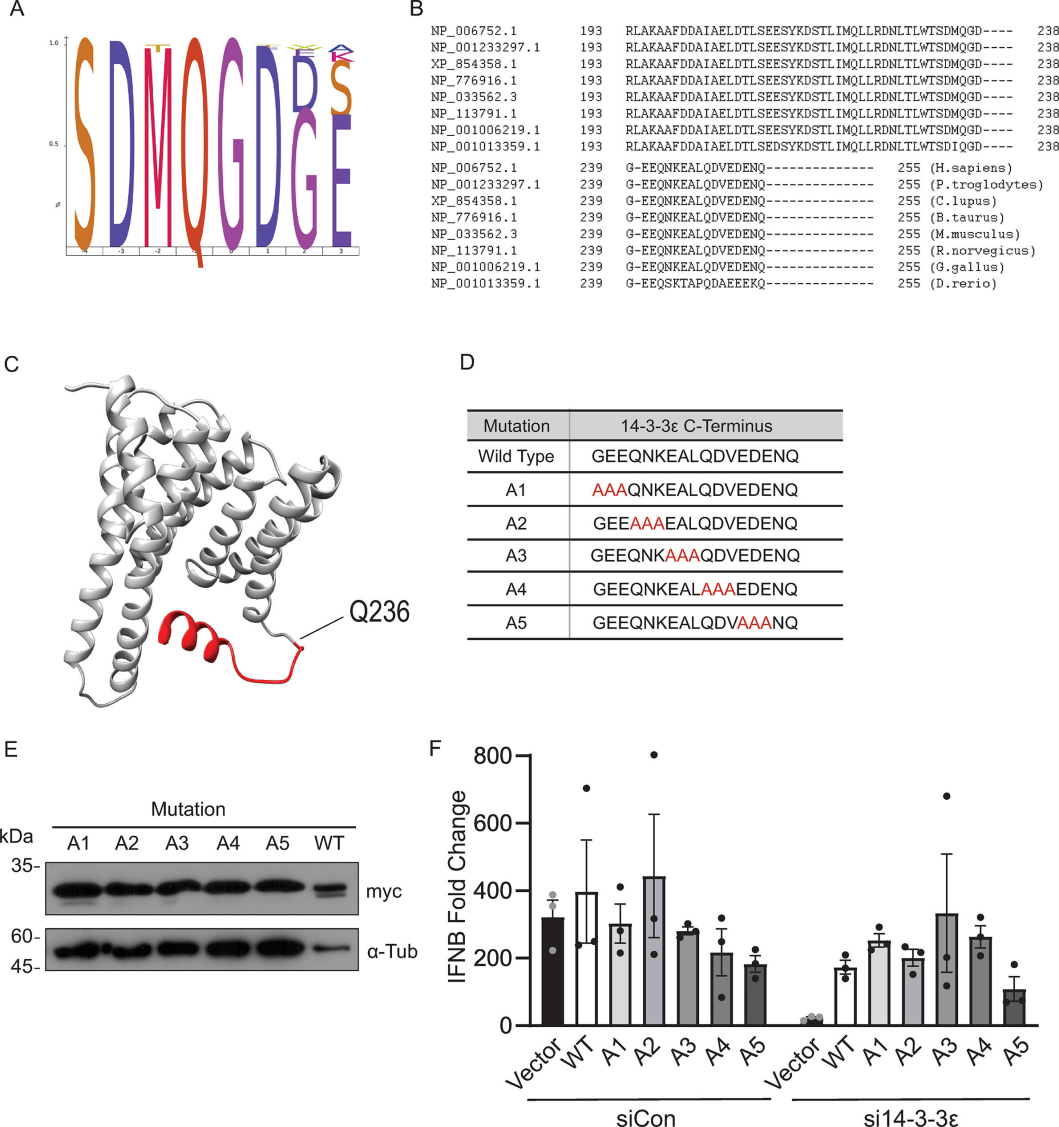
Key residues in the C-terminal region of 14-3-3ε for RIG-I signaling. (**A**) IceLogo analysis of the P4-P4’ TAILS-identified cleavage site of 14-3-3ε across several orders. Ninety-six Swiss-Prot manually annotated 14-3-3ε sequences were aligned and compared for sequence identity. (**B**) Residues 193–255 of 14-3-3ε from different species were aligned and compared for sequence identity. (**C**) *In silico* 3D structural model of 14-3-3ε generated using the RoseTTA fold and existing crystal structures (PDB: 6EIH). The 14-3-3ε 3C^pro^ cleavage site is indicated (Q236) and the C-terminal fragment is shown in red. (**D**) Scanning alanine mutagenesis of the 14-3-3ε C-terminus. G237–Q255 were sequentially mutated in codon triplets; mutations indicated in red. (**E**) Representative immunoblot of the scanning alanine mutants indicated in D. Cells were transfected with the indicated construct for 24 hours. (**F**) RT-qPCR of *IFNB* mRNA from cells treated with either siCon or si14-3-3ε for 24 hours, the indicated DNA for a further 24 hours, and poly(I:C) for 6 hours. Expression levels were normalized internally to *GAPDH*, and fold changes are relative to unstimulated control samples. *N* = 3. Statistical significance was analyzed using a two-way ANOVA.

Our results indicated that cleavage of 14-3-3ε by 3C^pro^ leads to the generation of a truncated N-terminal cleavage fragment that subsequently decreases RLR signaling and suggests that the C-terminal tail of 14-3-3ε may have a role in RLR signaling. The C-terminal tail is highly variable among the 14-3-3 family proteins and is predicted to be flexible and disordered (37). While highly variable between 14-3-3 protein family members, the C-terminus of 14-3-3ε is 100% conserved among mammals, and the 3C^pro^-induced QG cleavage site is 100% conserved (Fig. 4A and B). To date, a complete crystal structure of 14-3-3ε resolving the C-terminal tail has yet to be determined. It has been proposed that the charged residues in the C-terminal tail may regulate 14-3-3 function by interacting with the phospho-binding pocket of 14-3-3 proteins (24, 37, 38), though the detailed functions of the C-terminal tail of 14-3-3 proteins are not fully understood. Modeling the structure of the C-terminal domain of 14-3-3ε using the RoseTTA fold (39) *in silico* predicted a short α-helical fold (Fig. 4C).

To determine residues within the C-terminal region of 14-3-3ε that may be required for RLR signaling, we used a scanning alanine mutagenesis approach, systematically mutating three consecutive residues to alanine from G237 to Q255 (Fig. 4D). We overexpressed alanine-mutated 14-3-3ε constructs in A549 cells (Fig. 4E) treated with siCon or si14-3-3ε and then transfected with poly(I:C) to address the direct effects of C-terminal 14-3-3ε mutations on RLR signaling. In siCon-treated cells, the relative *IFNB* mRNA levels induced by poly(I:C) were similar across all constructs (Fig. 4F, left bar graph). We next determined the effects of these 14-3-3ε mutants in cells depleted of endogenous 14-3-3ε. As shown in Fig. 3G, expression of the WT 14-3-3ε in si14-3-3ε-transfected cells rescued poly(I:C)-stimulated *IFNB* mRNA expression as compared to vector-transfected cells (Fig. 4F, right bar graph). Similarly, expression of 14-3-3ε mutants (A1–A4) in the presence of si14-3-3ε exhibited a similar fold *IFNB* mRNA change to that of the WT 14-3-3ε expressing cells. By contrast, transfection of the 14-3-3ε A5 mutant construct in si14-3-3ε-treated cells did not fully rescue *IFNB* mRNA production, albeit this observation was not statistically significant (Fig. 4F). These results hinted that residues EDE251-253 within the C-terminal 14-3-3ε tail contribute to RLR signaling.

### 14-3-3ε mutations disrupt the RIG-I translocon

**Fig 5:**
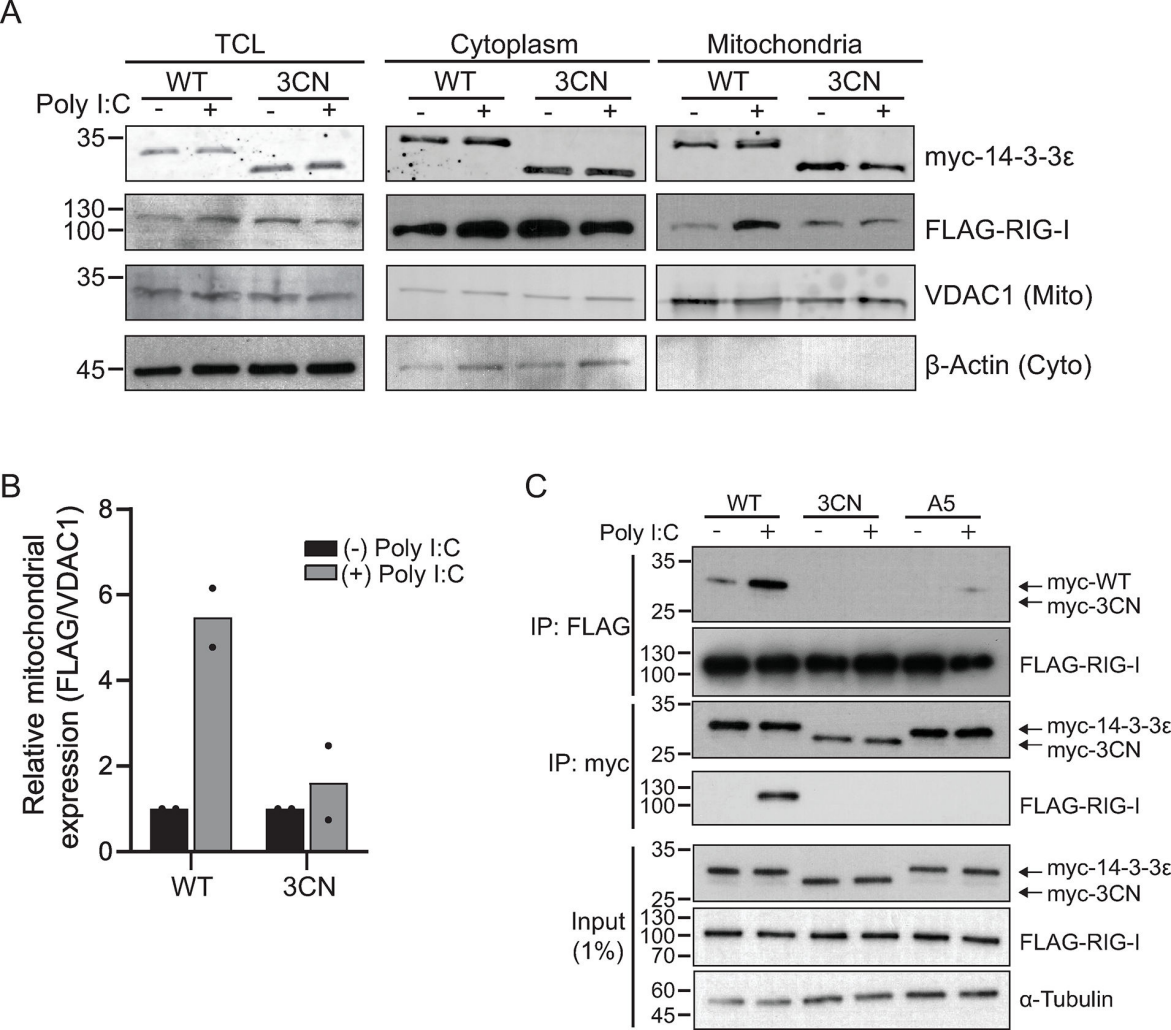
N-terminal 14-3-3ε overexpression disrupts the RIG-I translocon. (**A**) Immunoblot of myc-14-3-3ε, FLAG-RIG-I, VDAC1, and β-actin before and after subcellular membrane fractionation. Cell lysates were transiently transfected with FLAG-RIG-I and either myc-tagged wild type (WT) or 3CN 14-3-3ε constructs for 24 hours, then transfected with poly(I:C) for 6 hours. Lysates were subjected to membrane fractionation and analyzed via Western blot. TCL, total cell lysate; 5% input. *N* = 2. (**B**) Quantification of FLAG and VDAC1 band intensity in the mitochondrial fraction of (**A**). Bands were quantified using ImageJ, and FLAG band intensity was normalized to VDAC1 band intensity (*N* = 2). (**C**) Co-immunoprecipitation assay of cells transiently transfected with FLAG-RIG-I and either myc-tagged WT, 3CN, or A5 14-3-3ε constructs for 24 hours, then transfected with poly(I:C) for 6 hours. Lysates were precipitated using myc or FLAG antibodies that are covalently linked magnetic beads; 1% input (*N* ≥ 3).

Our results showed that overexpression of the 3CN 14-3-3ε disrupted the RLR signaling pathway upstream of TBK1 phosphorylation (Fig. 3K). We next asked whether expression of 3CN 14-3-3ε blocks RIG-I translocation to the MAVS at the mitochondria. To address this, we co-transfected FLAG-RIG-I and either the myc-tagged WT or 3CN 14-3-3ε constructs in HEK293T cells, and then monitored RIG-I localization in the mitochondrial and cytoplasmic fractions by immunoblotting. Robust co-expression of FLAG-RIG-I and myc-14-3-3ε in A549 cells was challenging, thus we turned to HEK293T cells that are more conducive for transfections. The mitochondrial marker voltage-dependent anion-selective channel protein 1 (VDAC1) and the cytoplasmic marker β-actin were enriched in their respective subcellular compartments, thus validating the membrane fractionation procedure (Fig. 5A). Consistent with previous reports (11), poly(I:C) treatment of cells expressing the WT 14-3-3ε resulted in an enrichment of FLAG-RIG-I in the mitochondrial fraction (Fig. 5A). Conversely, expression of 3CN 14-3-3ε did not lead to mitochondrial enrichment of FLAG-RIG-I compared to cells transfected with WT 14-3-3ε (Fig. 5A). Quantification of the immunoblots confirmed that the amount of FLAG-RIG-I localized to the mitochondria was decreased in cells expressing 3CN 14-3-3ε compared to cells expressing WT 14-3-3ε (Fig. 5B). These results suggested that expression of 3CN 14-3-3ε blocks signaling at or upstream of the RIG-I translocation step.

The RIG-I translocon complex involves several key protein-protein interactions that include at least 14-3-3ε, TRIM25, UFL1, and RIG-I (10, 11, 26). To determine whether 3CN 14-3-3ε can interact directly with RIG-I in poly(I:C)-stimulated cells, we performed co-immunoprecipitation assays in cells expressing FLAG-RIG-I and either the myc-tagged WT, 3CN, or A5 mutant 14-3-3ε proteins (Fig. 5C). Expression of tagged RIG-I and the WT 14-3-3ε in poly(I:C)-stimulated cells resulted in a detectable interaction between the two proteins (Fig. 5C). Specifically, poly(I:C)-stimulated cells displayed an enrichment of FLAG-RIG-I co-precipitating with the myc-tagged WT 14-3-3ε. In the reciprocal immunoprecipitation (using FLAG-RIG-I as bait), the WT myc-14-3-3ε co-precipitated with FLAG-RIG-I. Conversely, the myc-3CN 14-3-3ε protein failed to interact with FLAG-RIG-I in poly(I:C)-stimulated cells in pulldown assays using either tagged-protein as bait (Fig. 5C). These results suggested that the C-terminal tail of 14-3-3ε is important for interactions with RIG-I.

To further evaluate the role of the 14-3-3ε C-terminal tail in binding to RIG-I, we next asked whether mutations in the C-terminus of 14-3-3ε, specifically the A5 mutant (EDE251-253, which did not fully support RLR signaling; Fig. 4F), disrupt 14-3-3ε/RIG-I interactions. We co-transfected myc-14-3-3ε A5 and FLAG-RIG-I, stimulated with poly(I:C), and performed co-immunoprecipitation assays. Both myc-14-3-3ε A5 and FLAG-RIG-I were detected in cell lysates (Fig. 5C). Using myc-14-3-3ε A5 as a bait, FLAG-RIG-I was not detected in precipitates from cells stimulated with poly(I:C), similar to that observed with 3CN myc-14-3-3ε as bait (Fig. 5C). Likewise, the reciprocal pulldown using FLAG-RIG-I as bait displayed reduced interactions between myc-14-3-3ε A5 and FLAG-RIG-I (Fig. 5C). In summary, these results showed that the C-terminal tail of 14-3-3ε, specifically amino acids EDE251-253, plays a key role in the interaction between 14-3-3ε and RIG-I during poly(I:C) stimulation.

### N-terminal 14-3-3ε cleavage fragment modulates virus infection

**Fig 6:**
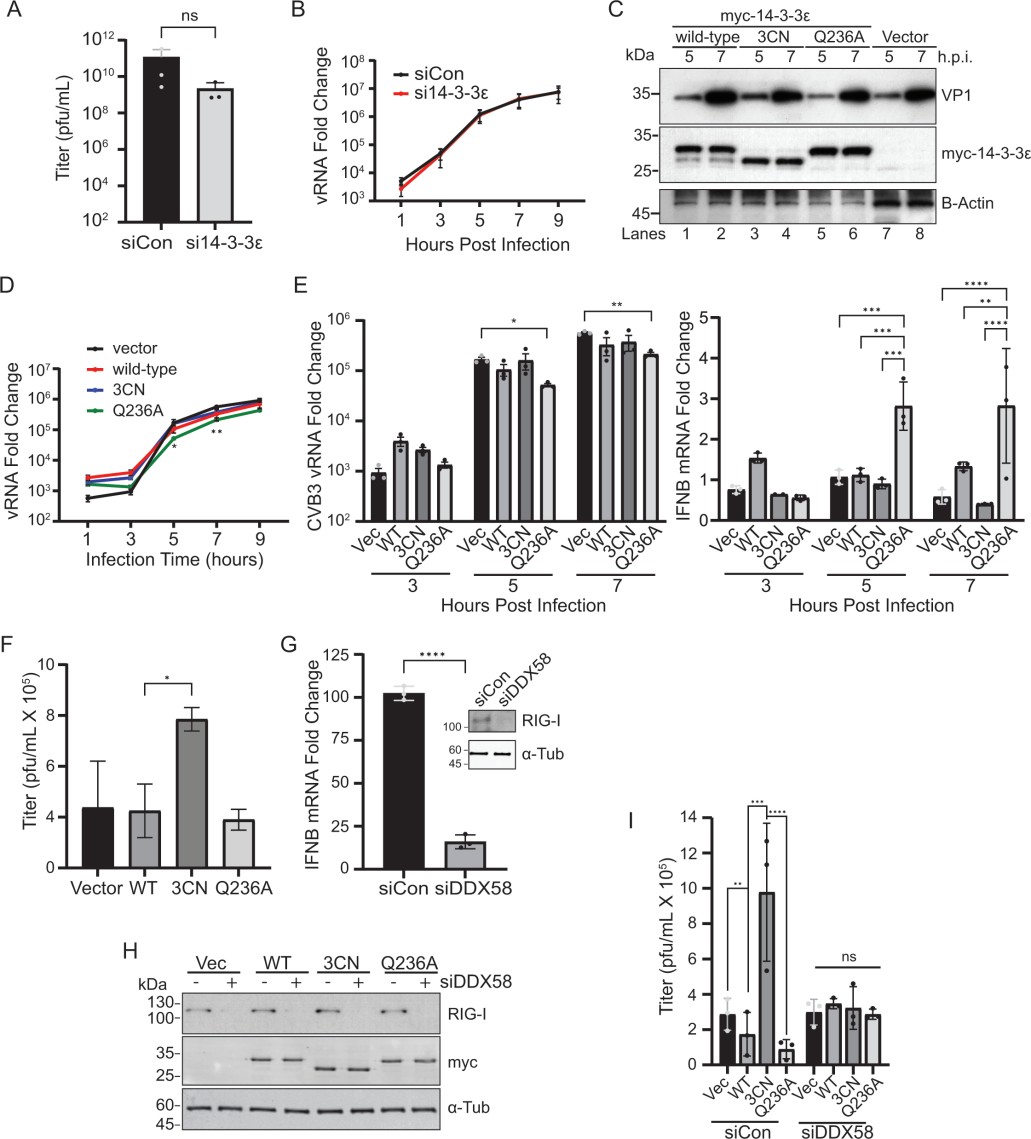
N-terminal 14-3-3ε modulates CVB3 infection. (**A**) Viral yield of CVB3-infected A549 cells transfected with either siCon or si14-3-3ε for 24 hours prior to infection (MOI = 0.01). Viral titers were determined via plaque assay (pfu - plaque forming units/mL) (*N* = 3, *P* = 0.404). (**B**) RT-qPCR analysis of CVB3 viral RNA from cells transfected with the indicated siRNA for 24 hours and subsequently infected with CVB3 for the indicated time (MOI = 5). Expression levels were normalized internally to *GAPDH* and fold changes are relative to uninfected control samples. (**C**) Immunoblot analysis of A549 cells transfected with the indicated myc-tagged or empty vector constructs for 24 hours, and subsequently infected with CVB3 for the indicated times post-infection (MOI = 5). (**D**) RT-qPCR analysis of CVB3 viral RNA from A549 cells transfected with the indicated DNA constructs for 24 hours and subsequently infected with CVB3 for the indicated time (MOI = 5). Expression levels were normalized internally to *GAPDH* and fold changes are relative to uninfected control samples. (**E**) Bar graph analysis of (**D**) RT-qPCR analysis of CVB3 viral RNA (left) and *IFNB* mRNA (right) at the indicated times; data generated from the same samples as (**D**). (**F**) Viral yield of CVB3-infected A549 cells (MOI = 0.01, 24 hours) that were transfected with the indicated DNA constructs. Cells were transfected for 24 hours prior to infection. Viral titers were determined (pfu/mL) via plaque assay. *N* ≥ 3. (**G**) RT-qPCR analysis of *IFNB* mRNA levels from A549 cells transfected with either siCon or siDDX58 for 48 hours and subsequently treated with poly(I:C) for 6 hours. Expression levels were normalized internally to *GAPDH* and fold changes are relative to unstimulated control samples. Inset: representative immunoblot of lysates treated with siCon or siDDX58 for 48 hours. *N* = 3. (**H**) Representative immunoblot of A549 cells transfected with the indicated siRNA for 48 hours and subsequently transfected with the indicated plasmid construct for 24 hours. (**I**) Viral yield of CVB3-infected A549 cells (MOI = 0.01, 24 hours) that were transfected with the indicated siRNA for 48 hours and the indicated plasmid construct for 24 hours. Viral titers were determined via plaque assay (pfu/mL). *N* = 3. *P < .05; **P < .005, ***P < .0005, ****P < .0001. Statistical tests used were Welch’s *t*-test (**E, H**) and one-way ANOVA (**B, C, D, F**).

We examined the role of 14-3-3ε in CVB3 infection by depleting 14-3-3ε via siRNA. Depleting 14-3-3ε resulted in, on average, a log-fold decrease in viral titer, albeit not statistically significant (Fig. 6A, *P* value = 0.404). We next monitored viral RNA levels in 14-3-3ε-depleted CVB3-infected cells. CVB3 viral RNA levels were similar in siRNA control versus si14-3-3ε treated cells, suggesting that 14-3-3ε does not affect replication (Fig. 6B). Taken altogether, these results indicated that depletion of 14-3-3ε does not adversely affect CVB3 infection.

We next addressed whether cleavage of 14-3-3ε at Q236 promotes enterovirus infection, and whether the resulting cleaved N-terminal fragment may have a role in infection. We overexpressed either the myc-tagged WT, 3CN, or cleavage-resistant mutant (Q236A) 14-3-3ε in A549 cells and then infected the cells with CVB3 at an MOI of 5. Immunoblotting showed detectable full-length WT, and Q236A myc-14-3-3ε at 5 and 7 h.p.i. and the slightly faster migrating N-terminal truncated 3CN myc-14-3-3ε (Fig. 6C). Notably, an N-terminal 14-3-3ε cleavage product was detected in cells expressing the WT but not the Q236A 14-3-3ε (Fig. 6C, compare lanes 1, 2–5, 6), thus further validating that cleavage by 3C^pro^ occurs between Q236↓G237. Interestingly, CVB3 viral protein 1 (VP1) expression consistently was slightly increased in cells expressing 3CN 14-3-3ε at 5 h.p.i. compared to the other 14-3-3ε-expressing cells (Fig. 6C, lanes 3, 4), thus hinting that overexpressing 3CN 14-3-3ε may promote infection. To examine this further, viral RNA levels measured by RT-qPCR were similar in CVB3-infected cells (MOI 5) expressing WT or 3CN 14-3-3ε over the course of infection compared to the vector control transfected cells (Fig. 6D and E—left bar graphs). By contrast, viral RNA levels in infected cells expressing Q236A 14-3-3ε were significantly reduced compared to other transfected cells (Fig. 6D and E—left bar graphs), thus suggesting that preventing the cleavage of 14-3-3ε reduces viral replication.

To assess whether this phenotype is linked to the cell’s innate immune response, we simultaneously measured *IFNB* mRNA levels by RT-qPCR in CVB3-infected cells (MOI 5) (Fig. 6E—right bar graphs). *IFNB* mRNA levels from CVB3-infected cells were normalized to that in mock-infected cells. Compared to vector control transfected cells, *IFNB* mRNA levels were relatively similar in CVB3-infected cells transfected with WT or 3CN 14-3-3ε constructs, in line that 3CN 14-3-3ε expression reduces RLR signaling (Fig. 6E). Furthermore, the reduced *IFNB* mRNA levels in CVB3-infected cells were not unexpected as several proteins such as RIG-I, MDA5, and MAVS in the RLR signaling pathway are targeted in CVB3-infected cells (40, 41). However, there was an approximately threefold increase in *IFNB* mRNA levels at 5 and 7 h.p.i in CVB3-infected cells expressing the cleavage-resistant Q236A 14-3-3ε (Fig. 6E). These results supported the conclusion that cleavage of 14-3-3ε acts to evade the cellular RIG-I response during infection.

To further examine these effects, we infected A549 cells expressing WT or mutant 14-3-3ε at an MOI of 0.01 to allow multiple rounds of infection and then monitored viral yield by plaque assay at 24 h.p.i. Cells transfected with the WT or the mutant Q236A 14-3-3ε did not stimulate nor reduce viral yield as compared to vector transfected control cells (Fig. 6F). By contrast, transfection of the 3CN 14-3-3ε construct resulted in a significant enhancement in viral yield, thus showing that the N-terminal 14-3-3ε 3CN fragment may act in a pro-viral manner (Fig. 6F).

To determine whether the primary role of 14-3-3ε cleavage by 3C^pro^ is to evade the host RIG-I response, we depleted endogenous RIG-I by siRNA transfection and monitored the effects of expressing WT or mutant 14-3-3ε in CVB3-infected cells. We first validated knockdown of endogenous RIG-I by immunoblotting (Fig. 6G) and showed that *IFNB* mRNA levels were reduced by ~85% in poly(I:C)-treated cells (Fig. 6G), thus demonstrating that RLR signaling is significantly abrogated in RIG-I-depleted A549 cells. In RIG-I-depleted cells, we expressed WT, 3CN, or Q236A 14-3-3ε, which were confirmed by immunoblotting (Fig. 6H), and then infected with CVB3 (MOI 0.01) and monitored viral yield at 24 h.p.i. In siRNA-control treated cells, transfection with the 3CN 14-3-3ε construct resulted in an increase in viral yield compared to vector and WT 14-3-3ε transfected cells, similar to that observed in Fig. 6F (Fig. 6I). By contrast, in RIG-I-depleted CVB3-infected cells, CVB3 viral yields were similar across all transfections (Fig. 6I). Notably, the increase in CVB3 viral yield observed in 3CN 14-3-3ε transfected siRNA-control cells was muted in RIG-I-depleted cells (Fig. 6I). Taken together, these results demonstrated that the expression of cleavage of the full-length 14-3-3ε and the resulting 3CN 14-3-3ε cleavage fragment have direct effects on CVB3 infection via RLR signaling.

## DISCUSSION

The RIG-I signaling pathway is a critical first-line host defense in detecting RNA virus infection and activating the antiviral IFN response (6). Highlighting the importance of this pathway are the diverse viral mechanisms that target RLR signaling to restrict IFN production (18, 20, 27, 28). 14-3-3ε is a key factor in the RIG-I translocon complex that signals to MAVS; however, the exact role of 14-3-3ε in the translocon complex is not well understood (11). In this study, we identified 14-3-3ε as a direct target of enterovirus 3C^pro^, which cleaves between Q236 and G237, thus providing insights into the key regions of 14-3-3ε important for RIG-I signaling. Expression of a 3C^pro^-mediated N-terminal 3CN 14-3-3ε cleavage fragment is unable to interact with RIG-I. We further mapped key residues in the C-terminal region of 14-3-3ε that are important for RIG-I signaling, thus providing a mechanistic rationale for cleavage by 3C^pro^ and release of the C-terminal tail. Finally, we showed that overexpression of the cleaved N-terminal 14-3-3ε fragment promoted CVB3 infection, supporting a pro-viral role for 14-3-3ε cleavage and the N-terminal fragment itself. We propose that strategic cleavage of 14-3-3ε by enterovirus 3C^pro^ contributes to evasion of the host antiviral RIG-I signaling pathway and promotes infection.

How does the 3C^pro^-mediated cleavage of 14-3-3ε disrupt and dampen RIG-I signaling? 14-3-3ε, along with RIG-I and TRIM25, form a translocon complex that plays a critical role in signaling from RIG-I to MAVS at the mitochondrial membrane (10, 11). Recently, it has been shown that 14-3-3ε is also ufmylated by UFL1 during RIG-I signaling, a further requirement for translocon formation and translocation (26). Indeed, post-translational modifications of RIG-I, MDA5, and their interaction partners play prominent roles in 14-3-3ε activation and regulation of RLR signaling (16). The dynamics and interplay of these factors are not fully understood. The N-terminal region of 14-3-3ε family members contains dimerization sites that mediate homo- or hetero-dimerization with itself or other 14-3-3 proteins, as well as a binding groove that facilitates binding to phosphorylated substrates (16, 37); however, previous studies have shown that 14-3-3ε likely binds to RIG-I in a phosphorylation-independent manner (11), suggesting that 14-3-3ε may interact with RIG-I via an atypical manner or indirectly via other translocon complex proteins. Although the truncated N-terminal 14-3-3ε fragment does not bind to FLAG-RIG-I (Fig. 5C), it is possible that it may also disrupt other interactions within the translocon complex, such as with TRIM25 and UFL1, or some combination of these. Furthermore, the cleaved N-terminal 14-3-3ε fragment may be endowed to bind to and be sequestered by other cellular proteins yet to be determined.

In general, the C-terminal tail of 14-3-3 family of proteins is poorly characterized (37). Our results reveal insights by identifying three C-terminal acidic residues (E251, D252, and E253) of 14-3-3ε that are important for 14-3-3ε/RIG-I interactions and in contributing to full RIG-I signaling (Fig. 4F and 5C), thus providing a mechanistic explanation as to why cleavage by 3C^pro^ after Q236 may be strategic to block signaling activity. Notably, it has been proposed that the disordered C-terminal region of 14-3-3 proteins may act in an autoinhibitory manner; Truong et al. (42) proposed that the C-terminal region is responsible for preventing promiscuity and fine-tuning specific protein-protein interactions. It follows that truncation of the C-terminal tail by the enterovirus 3C^pro^ may alter the interactions between 14-3-3ε and its ligands and disrupt downstream signaling pathways. It will be important to further elucidate precisely how the C-terminal domain contributes to 14-3-3ε interactions with RIG-I and other factors in the translocon complex, either directly or indirectly.

In enterovirus infections, several proteins in the RLR signaling pathway are cleaved and/or degraded, such as RIG-I (40), MDA5, and MAVS (41). As such, the incomplete cleavage of 14-3-3ε during infection may be sufficient, along with targeting the other RLR signaling factors, to ensure disabling the RLR signaling pathway. Here, our results point to the cleavage of 14-3-3ε as a key viral strategy to promote CVB3 infection. Notably, expressing the cleavage-resistant mutant Q236A 14-3-3ε reduced CVB3 viral RNA levels (Fig. 6C and D) and increased *IFNB* mRNA levels (Fig. 6D). Furthermore, the increase in CVB3 viral yield through expression of the cleavage fragment 3CN 14-3-3ε is RIG-I dependent (Fig. 6I). These results collectively indicate a dual viral strategy whereby both cleavage of the full-length 14-3-3ε and the resulting N-terminal 3CN 14-3-3ε cleavage fragment have direct effects on dampening RLR signaling in order to promote CVB3 infection. Given that 14-3-3ε normally acts as a chaperone protein for a wide range of signaling pathways and critical interactions with other proteins, 14-3-3ε may disrupt other cellular functions in such a way that restricts viral replication (25). Future studies of the interactomes of the full-length and N-terminal 14-3-3ε cleavage fragment under virus infection should provide insights into these mechanisms.

It is noted that expression of Q236A 14-3-3ε decreased CVB3 viral RNA levels concomitant with an increase in *IFNB* mRNA levels in CVB3-infected cells (MOI 5) (Fig. 6C and D), yet expression of Q236A 14-3-3ε did not decrease viral yield after multiple infection cycles (MOI 0.01, 24 hours) (Fig. 6D and G). In these transfections, endogenous 14-3-3ε is still present and subject to cleavage, resulting in expression of the 3CN cleavage fragment under CVB3 infection. Thus, it is likely that the accumulating 3CN 14-3-3ε cleavage fragment after multiple infection cycles leads to increased CVB3 viral yield (Fig. 6D and G), whereas this effect is not observed in a single infection cycle.

Targeting host cellular proteins by virally encoded proteases is an effective viral strategy to modulate host processes and evade antiviral responses (3 - 43 - 43). In enterovirus infection, targeting 14-3-3ε as well as several other factors in the RLR-signaling pathway is critical for productive infection and points to the importance of ensuring evasion of this pathway. It will be of interest to examine the roles of other 14-3-3 proteins that are cleaved under enterovirus infection. The recent advances in N-terminomics to identify host targets of viral proteases (3 - 43 - 43), both *in vitro* and in virus-infected cells, will provide insights into other antiviral factors that must be counteracted to promote virus infection.

## MATERIALS AND METHODS

### Cells and viruses

HEK293T, A549, and A549-Dual cells ( Invivogen) were cultured in Dulbecco’s Modified Eagle Medium (DMEM, Gibco 12100–046) supplemented with 10% fetal bovine serum and 1% penicillin/streptomycin at 37°C. A549 cells were kindly provided by Dr. Robert Hancock (University of British Columbia). Poliovirus (Mahoney type 1 strain; accession NC_002058.3) was generated from a pT7pGemPolio infectious clone. Poliovirus and coxsackievirus B3 stocks were propagated and titered in HeLa cells.

### Plasmids and transfections

GFP and myc-tagged 14-3-3ε were generated as follows: a G-block containing the full-length 14-3-3ε protein (IDT) was cloned into pEGFP-C1 (Addgene #2487) using XhoI and BamHI restriction sites. To generate truncated mutants via site-directed mutagenesis, the following primers were used: 5′-CTCGAGCTATGGATGATCGAGAGGATCTG-3′ (for 5′ end amplification of all constructs), 5′-GGATCCTTACTGCATGTCTGAAGTCCATAG-3′ (3CN), and 5′-GCTCTTCTTAGTCACCCTGCATGTCTGAAGT-3′ (CaspN). To swap the GFP tag with a myc tag, a double-stranded oligo (5′-GCTAGCGCCGCCATGGTGGAGCAAAAGCTCATTTCTGAAGAGGACTTGAGATCT-3′) was subcloned into the pEGFP-C1 plasmid using restriction sites NheI and BglII. To generate scanning alanine mutants, DNA fragments containing the desired mutations were synthesized and subcloned into the parent vector (myc-WT 14-3-3ε) using XhoI and BamHI restriction sites.

All DNA transfections were performed using Lipofectamine 2000 according to the manufacturer’s protocol (ThermoFisher, 11668019). Briefly, cells were seeded 24 hours before transfection to allow adherence. Transfection took place in antibiotic-free DMEM supplemented with 10% fetal bovine serum. All transfections were conducted for 16–24 hours prior to experimentation. For siRNA transfections, knockdowns were performed using DharmaFECT 1 (Dharmacon, T-2001–03) and a custom siRNA duplex (sense: 5′-CAUCUAAGAGAGAGGUUAAUU-3′; antisense: 5′-UUAACCUCUCUCUUAGAUGUU-3′) according to the manufacturer’s protocol. When applicable, cells were transfected with 10 µg/mL polyinosinic:polycytidylic acid (Invivogen).

### Virus infections

Poliovirus and coxsackievirus B3 viruses were incubated with cells at the indicated MOI for 1 hour in DMEM + 1% penicillin/streptomycin at 37°C. After adsorption, the media was replaced with complete DMEM (1× DMEM, 10% fetal bovine serum, 1% penicillin/streptomycin) and incubated for the designated time. For infections in the presence of zVAD-FMK (RD Systems, FMK001), zVAD-FMK was added to a final concentration of 50 µM 16 hours prior to infection, and virus-containing media was supplemented with 50 µM zVAD-FMK for the duration of infection.

### Proteomic data sets

The TAILS data set used was described in Jagdeo et al. (3) and is publicly available in the ProteomeXchange Consortium (proteomecentral.proteomexchange.org) database under the accession number PXD008718.

### Immunoblot analysis

Equal amounts of protein were resolved on an SDS-PAGE gel and subsequently transferred onto a polyvinylidene difluoride (PVDF; Millipore) membrane. The primary antibodies used were as follows: 1:2,000 α-Tubulin (ab4074); 1:1,000 14-3-3ε (CST #9635); 1:1,000 14-3-3 sampler kit (CST #9769T); 1:1,000 RIG-I (CST #3743); 1:2,000 c-Myc antibody (ThermoFisher MA1-980); 1:2,000 myc tag antibody (Bethyl A190-105A); 1:2,000 FLAG M2 (Millipore F1804); 1:1,000 VDAC1 (ab15895); 1:1,000 TBK1/NAK (CST #3504); 1:1,000 phospho-TBK1/NAK (CST #5483); 1:1,000 hnRNP M (sc-134360); 1:3,000 VP1 (Dako); 1:1,000 β-Actin (ab8224); 1:1,000 PARP (CST #9542); and 1:2,000 GFP (Roche 11814460001).

### *In vitro* cleavage assay

*In vitro* cleavage assays were performed as described (4). Briefly, lysates were resuspended in cleavage assay buffer (20 mM HEPES, 150 mM KOAC, 1 mM dithiothreitol) in the presence of protease inhibitors (ThermoFisher, cat. no. 78440). Lysates were incubated with WT or catalytically inactive poliovirus 3C^pro^ (C147A) and the reaction was quenched after the indicated time using SDS-PAGE loading buffer.

### RT-qPCR

RT-qPCR was performed using total cellular cDNA. Briefly, cells were harvested in 1 mL Trizol (ThermoFisher, 15596018) and total cellular RNA was isolated according to the manufacturer’s protocol. RT-qPCR was performed using the NEB Luna Universal One-Step RT-qPCR Kit (#E3005L) using 20 ng total RNA. The following primers were used for analysis: GAPDH (5′-GGTGGTCTCCTCTGACTTCAACA-3′, 5′-GTTGCTGTAGCCAAATTCGTTGT-3′), IFNB (5′-TAGCACTGGCTGGAATGAGA-3′, 5′-TCCTTGGCCTTCAGGTAATG-3′), and CVB3 RNA (5′-GCACACACCCTCAAACCAGA-3′, 5′-ATGAAACACGGACACCCAAAG-3′).

### Membrane fractionation

Cell lysates were separated into cytosolic or mitochondrial fractions using the BioVision Mitochondria/Cytosol Fractionation Kit (BioVision #K256) according to the manufacturer’s protocol. Briefly, cells were lysed by passing cells through a 25 g needle 25–30 times. Unbroken cells and nuclei were cleared by centrifugation at 700 g until no pellet was observed. The samples were then spun at 10,000 g and the supernatant was saved as the cytosolic fraction. The remaining pellet was resuspended in mitochondria extraction buffer as the mitochondrial fraction.

### Co-immunoprecipitation

Cells were lysed in lysis buffer containing 1% Triton-X, 150 mM NaCl, and 1X HALT protease inhibitors (Thermo Fisher #78429) on ice for 20 minutes before clarification. Five hundred micrograms to one milligrams of cell lysate was then incubated with either Pierce Anti-c-Myc Magnetic Beads (Thermo Fisher #88842) or Anti-FLAG M2 Magnetic Beads (Millipore Sigma, M8823) for 1 hour. Beads were then separated using a magnetic stand and washed twice with ice-cold lysis buffer (NaCl adjusted to 300 mM) and twice with ice-cold dH_2_O. Antibody-bound protein was eluted at 95°C for 5 minutes, and the supernatant was collected for downstream analysis.

### Plaque assay

Plaque assays were performed as follows: a confluent monolayer of HEK293T cells were infected with a dilution series (10^−1^ to 10^−7^) of virus absorbed in a minimum volume of serum-free media for 1 hour. The inoculum was subsequently removed and the monolayer overlaid with 1% methylcellulose in DMEM for 72 hour. The overlay was then discarded, and the cells were stained for 15 minutes at room temperature in 1% crystal violet (wt/vol) and 50% methanol (vol/vol).

### Cell viability assay

MTT assays were performed using the CyQUANT Cell Viability Assay Kit (ThermoFisher, V13154) according to the manufacturer’s instructions. Briefly, A549 cells were seeded in a 96-well plate for 24 hours prior to performing the assay. Control wells with no cells were used for subtracting the background signal, and a serial dilution of cells (10^3^ to 10^7^) was used to generate a standard curve.

### Statistical analysis

All graphs were created and statistical testing performed using GraphPad Prism 9.0. * *P* < 0.05; **P < 0.005; ***P < 0.0005; ****P < 0.00005. For RT-qPCR data, significance was determined using either a Welch’s *t*-test, a one-way ANOVA, or a two-way ANOVA as appropriate based on the data set. The exact statistical test used for each analysis is indicated in the relevant figure legend.

## Data Availability

Further information and requests for resources and reagents can be directed to Eric Jan (ej@mail.ubc.ca).
